# Electrical Activation in the Coronary Sinus Branches as a Guide to Cardiac Resynchronisation Therapy: Rationale for a Coordinate System

**DOI:** 10.1371/journal.pone.0019914

**Published:** 2011-08-08

**Authors:** Christoph Scharf, Nazmi Krasniqi, Jens Hellermann, Mariette Rahn, Gabor Sütsch, Corinna Brunckhorst, Firat Duru

**Affiliations:** 1 Division of Pacing and Electrophysiology, Clinic for Cardiology, Cardiovascular Center, University Hospital Zurich, Zurich, Switzerland; 2 Cardiology, Clinic im Park, Zurich, Switzerland; 3 Center for Integrative Human Physiology, University of Zurich, Zurich, Switzerland; Cornell University, United States of America

## Abstract

**Background:**

For successful cardiac resynchronisation therapy (CRT) a spatial and electrical separation of right and left ventricular electrodes is essential. The spatial distribution of electrical delays within the coronary sinus (CS) tributaries has not yet been identified.

**Objective:**

Electrical delays within the CS are described during sinus rhythm (SR) and right ventricular pacing (RVP). A coordinate system grading the mitral ring from 0° to 360° and three vertical segments is proposed to define the lead positions irrespective of individual CS branch orientation.

**Methods:**

In 13 patients undergoing implantation of a CRT device 6±2.5, (median 5) lead positions within the CS were mapped during SR and RVP. The delay to the onset and the peak of the local signal was measured from the earliest QRS activation or the pacing spike. Fluoroscopic positions were compared to localizations on a nonfluoroscopic electrode imaging system.

**Results:**

During SR, electrical delays in the CS were inhomogenous in patients with or without left bundle branch block (LBBB). During RVP, the delays increased by 44±32 ms (signal onset from 36±33 ms to 95±30 ms; p<0.001, signal peak from 105±44 ms to 156±30 ms; p<0.001). The activation pattern during RVP was homogeneous and predictable by taking the grading on the CS ring into account: (% QRS) = 78−0.002 (grade−162)^2^, p<0.0001. This indicates that 78% of the QRS duration can be expected as a maximum peak delay at 162° on the CS ring.

**Conclusion:**

Electrical delays within the CS vary during SR, but prolong and become predictable during RVP. A coordinate system helps predicting the local delays and facilitates interindividual comparison of lead positions irrespective of CS branch anatomy.

## Introduction

Left ventricular dyssynchrony has been shown to correlate with the time delay needed for electrical activation from the septum to the free wall of the left ventricle [1]. The magnitude of improvement in synchrony after cardiac resynchronisation therapy (CRT) correlates with the electrical delay measured at the implanted coronary sinus (CS) lead during sinus rhythm [2]. In addition, the response to CRT correlates with the electrical delay measured between the implanted coronary sinus (CS) lead and the right ventricular (RV) lead, the interlead delay [3], [4]. As a consequence, implanting the CS electrode at the site of latest electrical activation of the individual patient seems reasonable. Although the area of latest electrical activation during SR has been mapped endocardially [5], [6], it may not correspond to epicardial activation times within the CS. In addition, the limited number and locations of CS branches accessible for positioning the CS lead further restricts applicability of preimplant mapping results. Finally, during CRT, the region with latest activation during SR might not correspond to the region with the greatest electrical separation during RV pacing. In other words, simultaneous RV pacing from the implant lead might not match with preimplant mapping results during SR.

The purpose of this study was to predict electrical activation within different CS branches during CRT implantation using a three dimensional imaging system and compare SR and RV pacing in different types of conduction block. Given the high interindividual variability of CS branch morphology, the lead positions during mapping are reported by segments [7]. We have refined this concept by proposing a coordinate system with gradation of the mitral ring to predict the site of latest electrical activation to guide CS lead implantation.

## Methods

The subjects of this study were 13 consecutive male patients referred for implantation of a CRT device (table 1). All patients underwent implantation of the atrial lead and the RV lead at the apical septum. Then, CS mapping was performed using the implant electrode, as a clinical protocol in order to maximize electrical separation between the RV and left ventricular (LV) leads.

**Table 1:** Patients' characteristics.

*Total number of patients*	*13*
sex	13 male
Age (years)	67±7
EF	0.25±0.046
Ischemic CMP	9 (69%)
Nonischemic CMP	4 (31%)
NYHA Class I–II	4 (31%)
NYHA Class II–III	8 (62%)
NYHA Class III–IV	1 (7%)
**Medication:**	
B Blocker	11 (85%)
ACE Inhibitor	13 (100%)
Loop diuretics	13 (100%)

The clinical characteristics of the patients are summarized. EF = ejection fraction, CMP = Cardiomyopathy, NYHA = New York Heart Association Class, ACE = Angiotensin Converting Enzyme.

The study was approved by the institutional ethics committee and all patients gave informed consent.

### Electrical mapping

Electrograms at each position were saved on an electrophysiologic recording system (Bard EP, Lowell, MA), filtered at 50–200 Hz and saved for analysis during SR and RV pacing at 100 bpm. The pacing rate was 100 bpm in all but one initial patient, who was mapped during baseline pacemaker rate of 60 bpm. The delay from the beginning of QRS activation or the pacing spike to the onset and to the peak of the local electrical signal recorded on the CS electrode, was measured at each position. Depending on the type of lead, the electrograms were recorded in bipolar (50%) and unipolar (50%) mode (Table 1).

Lead positions were filmed fluoroscopically in two projections (AP and LAO 30°). In addition, each position was viewed and saved on a nonfluoroscopic imaging system (LocaLisa®, Medtronic, MN). This three-dimensional imaging system localises positions by using 6 skin electrodes for external application of a high frequency transthoracic electric field [8]. The voltage gradient between the CS lead electrode and the skin patches is then used to calculate the electrode position in three dimensions. The atrial lead with active fixation was used as the reference electrode in 13 patients and the RV lead in one patient without atrial lead.

### Definition of a coordinate system

**Figure 1:**
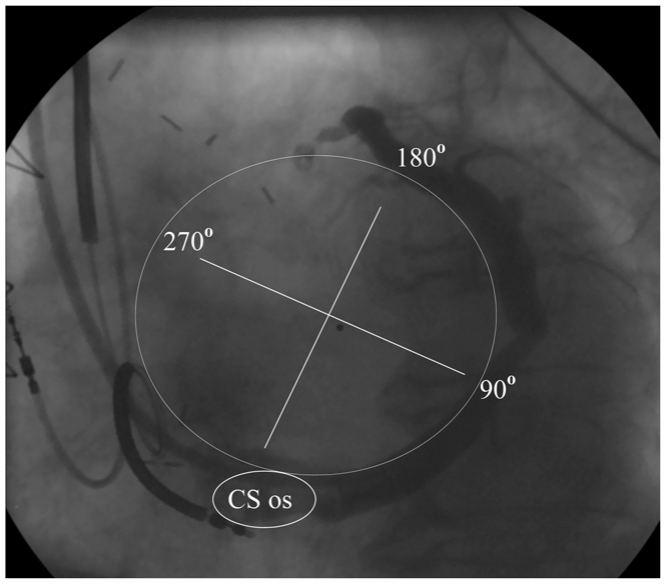
CS lead position is defined by a coordinate system using the projection on the mitral ring in one plane. The grading begins at the CS ostium and reaches 180° at the turnaround point, when the spatial distance to the CS ostium begins to decrease. With this grading system the position of a CS lead can be defined irrespective of the actual morphology of CS branches.

Positions within the CS and its branches were defined by their location in projection to the CS ring in the mitral annulus plane. The ring was defined by the CS ostium, beginning with 0°, and the turnaround point from posterior to anterior positions as 180° (Fig. 1). As the coronary sinus passes from posterior to anterior, the direction of the grading system is in the anticlockwise rotation. In the third vertical dimension segments were defined as basal, middle and apical [9]. Each position was located on the coordinate system by two investigators analyzing fluoroscopic images together with the orientation of the points in the nonfluoroscopic imaging.

### Statistical analysis

Continuous variables are expressed as means ± SD and were compared using student's t-test between groups. Correlations were calculated using Pearson's coefficients; quadratic regression was used to describe electrical delay activation times during RV pacing. In addition, an analysis using weighted parameters was performed to account for different number of positions per patient. Given the good correlation with a quadratic function, we assumed a nonlinear model of the association between the delay to the peak of the local electrical signal, which we expressed as percentage of total paced QRS duration, and the degree on the CS ring. The Gauss-Newton method with step halving was used as iterative technique, using least-squares. The Newton-Raphson method was used for finding profile confidence limits of the graphically estimated parameters. All analyses were done with the statistical software package JMP® version 5.0.1.2 (SAS Institute Inc., Cary, NC) and SPSS for windows (SPSS 12.0.1, Chicago, IL). For all tests, a p value <0.05 indicated statistical significance.

## Results

There were 13 consecutive patients, all male, with a mean age of 62±7 years (Table 1). Coronary artery disease was present in 9 patients with scar mostly in the anterior region. Only one patient had a posterior scar (Table 2). Four patients had moderate to severe mitral regurgitation. The mean ejection fraction was 22±6% (range 10–35%) with a LV enddiastolic diameter of 77±4 mm on echocardiography.

**Table 2:** The longest electrical delay during SR and RV pacing.

										Measurements during SR					Measurements during RV pacing				
ID	age	positions	ECG	scar	CS lead	polarity	spacing	RV lead	spacing	QRS	grade	seg	onset	peak	QRS	grade	seg	onset	peak
1	64	3	RBBB, LAHB	A	M 4193	U		M 6944	10 mm	146 ms	150°	2	21 ms	68 ms	199 ms	150°	2	167 ms	183 ms
2	73	5	RBBB, LAHB	A,S,I	M 4194	B	11 mm	M 6944	10 mm	192 ms	180°	1	54 ms	94 ms	240 ms	150°	2	154 ms	176 ms
3	62	5	LBBB	A,I	G 4525	B	11 mm	G 0165	12 mm	218 ms	180°	2	113 ms	182 ms	220 ms	180°	2	117 ms	166 ms
4	50	9	LBBB	A,S,I	M 4193	U		M 6944	10 mm	170 ms	225°	2	92 ms	160 ms	202 ms	180°	2	131 ms	190 ms
5	57	3	LBBB	A,I	M 4194	B	11 mm	M 6944	10 mm	182 ms	90°	3	64 ms	112 ms	224 ms	180°	1	68 ms	136 ms
6	65	7	LBBB	none	M 4194	B	11 mm	M 6944	10 mm	193 ms	120°	2	97 ms	105 ms	196 ms	105°	1	133 ms	171 ms
7	77	4	LBBB	P,I	M 4193	U		M 6944	10 mm	240 ms	90°	3	8 ms	180 ms	282 ms	195°	1	100 ms	206 ms
8	65	5	LBBB	none	M 4193	U		M 6944	10 mm	198 ms	180°	1	82 ms	186 ms	240 ms	180°	2	28 ms	172 ms
9	64	5	LBBB	A,L,I	M 4193	U		M 6944	10 mm	154 ms	195°	1	16 ms	98 ms	250 ms	130°	1	48 ms	220 ms
10	59	11	AVB	A,I	M 4194	B	11 mm	M 6944	10 mm	-	-	-	-	-	238 ms	165°	1	164 ms	208 ms
11	60	8	AVB	A,I	M 4193	U		M 6944	10 mm	-	-	-	-	-	235 ms	150°	1	131 ms	185 ms
12	64	4	AVB	A	M 4193	U		SJM1452T	12 mm	-		-	-	-	233 ms	120°	1	157 ms	215 ms
13	61	7	AVB	none	M 4194	B	11 mm	M 6944	10 mm	-	-	-	-	-	204 ms	190°	1	78 ms	160 ms

Electrocardiographic conduction, scar and sites of the longest electrical delay during SR and RVP. Patient Nr. 6 showed a relatively homogenous conduction pattern during RVP with a delay at 150° only 3 ms shorter than on 105°. RBBB, right bundle branch block; LBBB, left bundle branch block; LAHB, left anterior hemiblock; scar location A = anterior, I = inferior, S = septal, M = Medtronic, G = Guidant, U = unipolar, B = bipolar, RV = right ventricular, seg = vertical segment (1 = basal, 2 = middle, 3 = apical).

The CRT devices were implanted and tested during a total procedure time of 170±30 min (range 120–200 min), which included mapping inside the CS for 27±10 min. The mean fluoroscopy time was 28±10 min total was used for lead implantation. CS cannulation, venograms and filming was performed for a mean of 6±3 positions per patient in two projections (median 5, IQ 4.5–8).

### CS mapping along mitral ring

A total of 79 positions were mapped during RV pacing at 600 ms and during SR in patients with AV nodal conduction (Table 3). During SR, a moderate correlation between the peak electrical delay within the CS branches and total QRS duration was observed (Pearson's r = 0.51, p<0.001), but no relation to the position, the degree of lateral position on the mitral ring, nor the vertical position (basal segments vs. apical) was found (Table 3). In patients with left bundle branch block (LBBB), a heterogeneous activation pattern was found during SR with latest activations ranging from 90° to 225° on the mitral ring (Table 2) and often increasing delays towards the apical segments (Table 3). The delay of the peak of the electrical activation was longer in patients with LBBB versus right bundle branch block (RBBB) (signal onset 36±35 ms vs. 43±25 ms, ns; signal peak 108±45 ms vs. 80±12 ms, p = 0.002, respectively).

**Table 3:** Electrical delays during RVP and SR.

RVP all patients		0–90°		Nr	91–180°		Nr	>181°		Nr
		Delays in ms	Delay% of QRS		Delays in ms	Delay% of QRS		Delays in ms	Delay% of QRS duration	
**basal**	**onset**	89±24 ms	39±9%	4	114±36 ms	50±17%	16	89±15 ms	39±5%	11
	**peak**	122±18 ms	53±3%	4	176±30 ms	76±13%	16	159±24 ms	70±8%	11
**middle**	**onset**	82±12 ms	34±3%	4	106±23 ms	48±15%	17	80±16 ms	35±6%	13
	**peak**	147±46 ms	60±8%	4	164±16 ms	74±10%	17	138±27 ms	61±11%	13
**apical**	**onset**	68±43 ms	28±7%	6	96±34 ms	44±15%	5	74 ms	31%	1
	**peak**	142±51 ms	58±14%	6	150±23 ms	68±11%	5	95 ms	40%	1

SR in LBBB pts		0–90°			91–180°			>181°		
**basal**	**onset**	13±4 ms	6±1%	2	26±27 ms	13±13%	6	16±12 ms	8±5%	7
	**peak**	122±42 ms	55±10%	2	93±38 ms	47±17%	6	80±28 ms	43±14%	7
**middle**	**onset**	16±14 ms	8±8%	3	40±38 ms	21±20%	9	19±31 ms	11±18%	9
	**peak**	137±44 ms	59±13%	3	112±50 ms	56±23%	9	80±47 ms	43±27%	9
**apical**	**onset**	35±25 ms	17±15%	3	44±40 ms	22±20%	3	N.A.		
	**peak**	135±28 ms	61±8%	3	135±35 ms	66±16%	3	N.A:		

SR in RBBB pts		0–90°			91–180°			>181°		
**basal**	**onset**	N.A.			38±23 ms	21±10%	2	N.A.		
	**peak**	N.A.			81±18 ms	48±2%	2	N.A.		
**middle**	**onset**	N.A.			40±42 ms	22±23%	2	10 ms	5%	1
	**peak**	N.A.			78±17 ms	41±9%	2	66 ms	34%	1
**apical**	**onset**	68 ms	35%	1	50±25 ms	26±13%	2	N.A.		
	**peak**	74 ms	39%	1	80±8 ms	42±4%	2	N.A.		

Electrical delays in SR compared to RVP. SR = sinus rhythm, RVP = right ventricular apical pacing, RBBB = right bundle branch block, LBBB = left bundle branch block. Nr = number of measurements.

**Figure 2:**
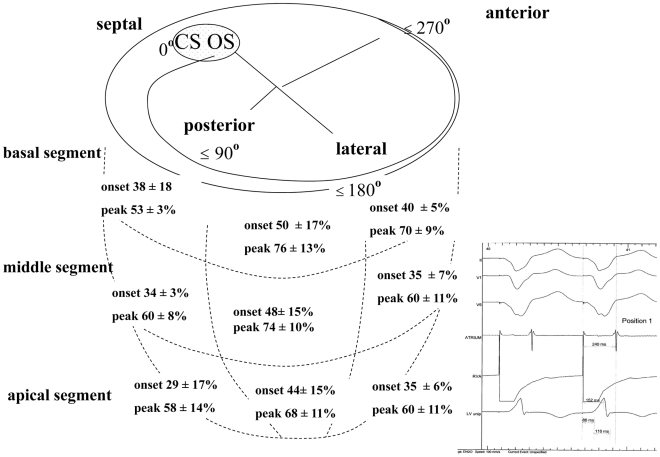
The electrical activation is measured during pacing from the implanted RV electrode. A) The onset and peak of the signal are referenced to the RV pacing spike and are depicted in quarters of the CS ring and cardiac segments. The greatest delay is found between 90° and 180° at the basal segment. In the lower left quadrant an example of the measurements is shown as an original tracing with 3 surface ECG leads, the atrial lead, the right ventricular pacing lead and the CS electrode. The QRS width is 240 ms, the peak delay 152 ms and the onset of the delay 86 ms after the pacing spike.

**Figure 3:**
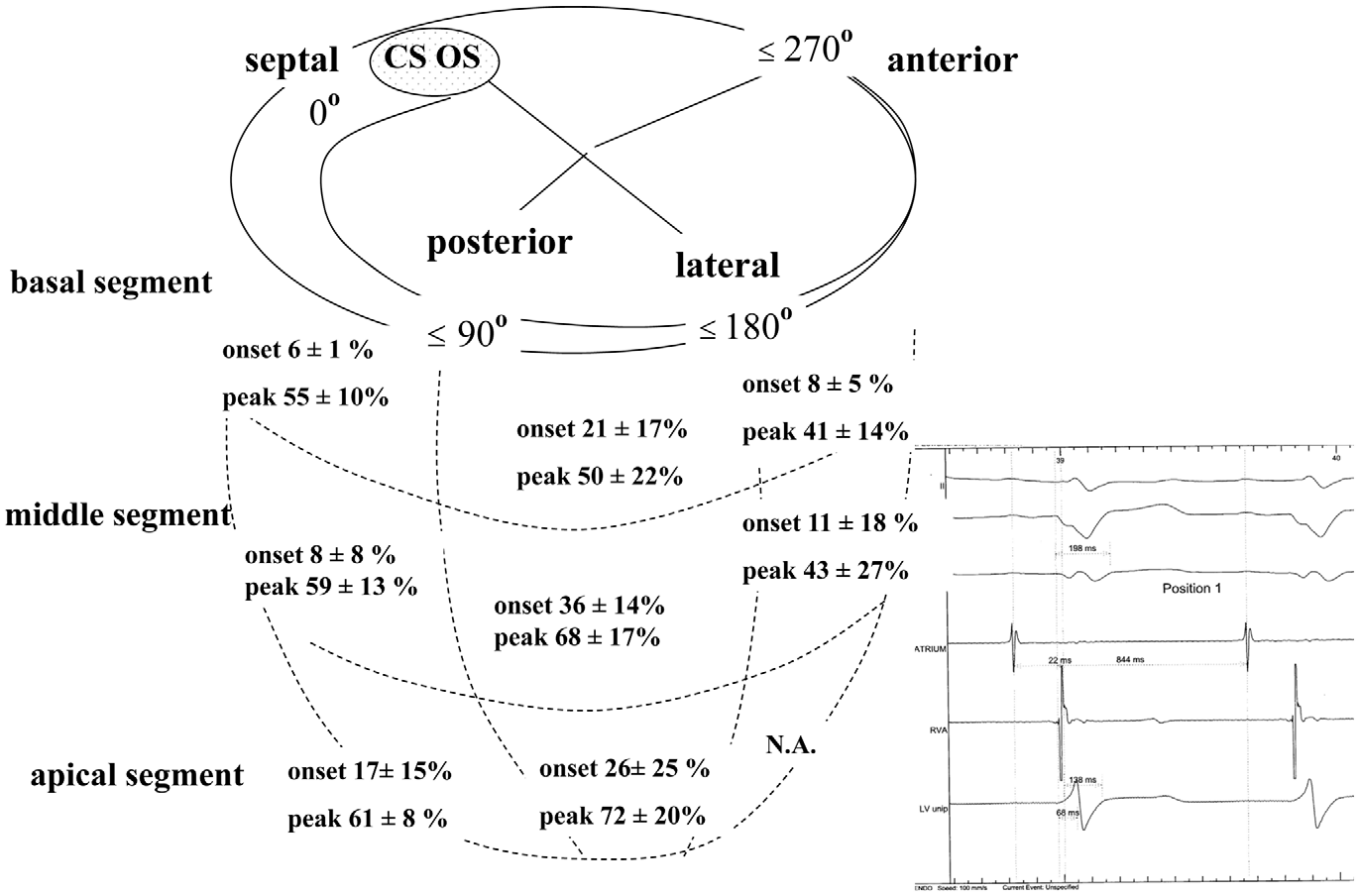
The electrical delays in sinus rhythm in LBBB patients, expressed as percentage of sinus QRS duration: at each CS lead position the corresponding delays during SR and RV pacing were subtracted from each another. In the lower left quadrant an example of the measurements is shown as an original tracing during sinus rhythm at the identical position as in figure 2. The QRS width is 198 ms, the peak delay 68 ms and the onset of the delay 22 ms after the pacing spike. It becomes evident, that the measurements in sinus rhythm show a large variation compared to RV pacing.

**Figure 4:**
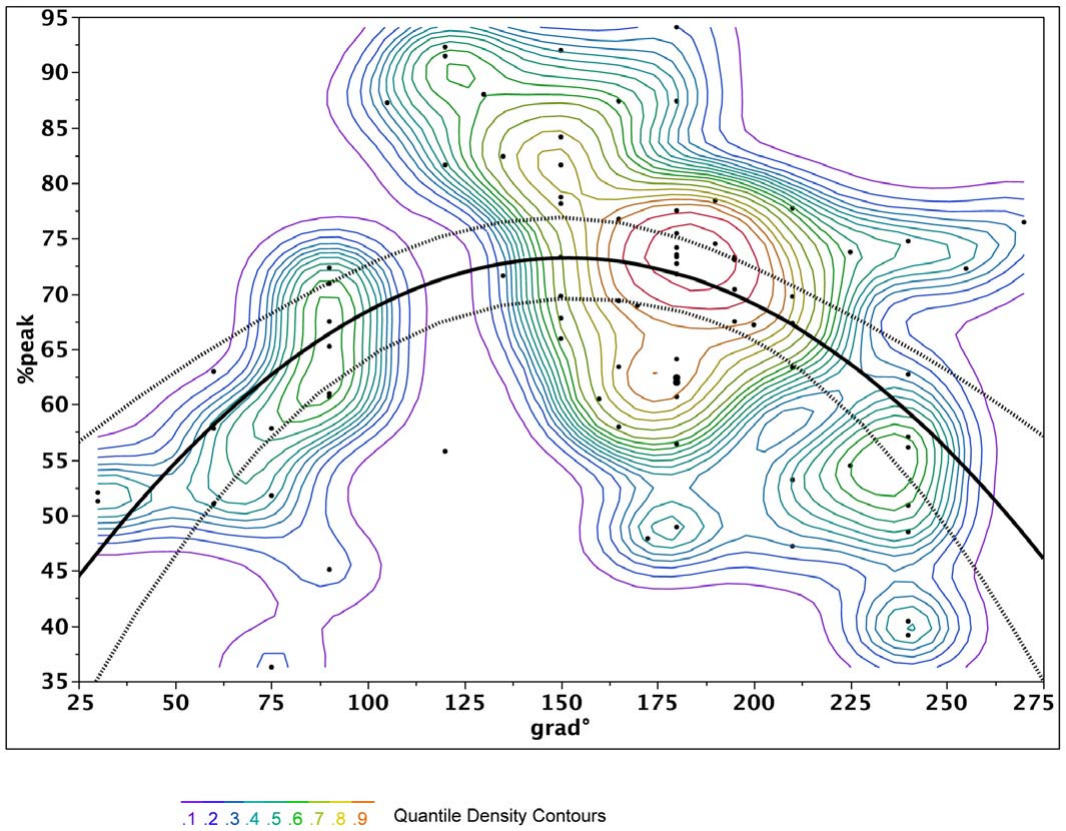
Graphical display of the association between peak signal delay expressed as percentage of total paced QRS duration and the degree on the CS ring, with 95% confidence intervals. Similar curves are found with onset of signal. The parabolic curve can be expressed as (% of QRS) = 78−0.002 (grade−162)^2^, p<0.0001. Additionally, a quintile density contour plot has been superimposed to account for different number of positions per patient. The contour lines are quintile contours in 10% intervals. This means that about 10% of the points are below the lowest contour, 10% are below the next contour, and so forth. The highest contour has 90% of the points below it. The solid line represents the fitted curve for the prediction of electrical delays (QRS peak), the dashed lines represent the 95% CI interval.

In contrast, RV pacing equalized electrical delays to a homogenous pattern (Table 2, Figures 2 and 3). The latest signals were found in the basal segment in the posterolateral region around 162° and increased towards the septum and anteriorly, irrespective of previous conduction system disease. As a result, the degree of peak signal delay expressed as percentage of total paced QRS duration was predictable by the following formula: delay to signal peak (% of QRS) = 78−0.002 (grade−162°)^2^, p<0.0001, which indicates that 78% of the QRS duration can be expected as local peak delay at 162° on the CS ring. A similar but less significant relationship was noted for the onset of the electric signal (p = 0.038). These results were confirmed by a weighted analysis accounting for the different number of positions per patient and are illustrated in Figure 4.

### CS mapping results in the vertical direction

During SR, a heterogeneous activation pattern was found in the vertical direction (table 3). During RV apical pacing a different activation sequence was found in the posterior region (0–90°; basal first), the posterolateral (91°–180°; basal latest) and the anterolateral (<180°; basal latest , table 3).

### SR versus RV pacing

In 9 patients (64%) with intact AV nodal conduction, the delays in SR were compared to RV pacing at each position (Table 3, Figures 2 and 3). The cycle length in SR was 825±161 ms and during RV pacing 593±17 ms (one patient had to be paced faster than 600 ms to overcome intrinsic SR). The surface QRS duration during SR was 186±17 ms and increased to 211±16 ms during RV pacing. Pacing from the implanted RV electrode increased electrical delays in the CS significantly in all patients (signal onset 36±33 ms vs. 95±30 ms, p<0.001, signal peak 105±44 ms vs. 156±30 ms, p<0.001). This effect was more pronounced in patients with RBBB (increase of signal onset by +93±29 ms, signal peak by +81±28 ms) than in LBBB patients (increase in signal onset by 53±28 ms, signal peak by 48±40 ms). Nevertheless, in patients with LBBB there was a considerable and heterogeneous prolongation of electrical delays during RV pacing compared to SR. The pacing-induced increase in electrical delay was considerable over the segments and rose up to 60% of the QRS duration. As a control group, patients with AV-block were paced at an intrinsic cycle length of 942±120 ms and 600 ms, which increased their onset of signals by 7±20 ms and peak delay by 6±30 ms (ns).

### Follow-up

The response to CRT was assessed clinically and by routine echocardiography after a mean follow up of 9±2 months (Table 4). Among the 13 patients 6 (46%) improved clinically and by basic echocardiographic parameters (increase in ejection fraction by at least 5% and decrease of LV diameters). Overall, there was no correlation between the clinical response and the degree of electrical delay at the final electrode position in this small patients group. The peak interlead delay was 179.2±22 ms in responders vs. 163±32 ms in nonresponders (p = 0.6) during RV pacing (78±10% vs. 72±13% of QRS duration, p = 0.6). The electrical delay to the LV electrode in sinus rhythm was 130±52 ms vs. 123±55 ms, respectively (p = 0.8).

**Table 4:** Echocardiographic findings at baseline and during follow-up.

ID	EF %pre	EF %post	LVEDD(mm)pre	LVEDD(mm)post
1	35	34	68	69
2	30	28	62	64
3	20	22	60	63
4	23	29	73	71
5	30	22	74	74
6	24	38	63	54
7	23	29	73	71
8	25	22	79	86
9	20	26	66	62
10	28	38	76	75
11	22	18	69	70
12	27	48	53	51
13	20	22	-NA	NA-

Left ventricular dimensions missing in patient 13. Routine echocardiographic findings at baseline and during follow up. There were 7 responders as defined by improvement of symptoms, increase in ejection fraction by at least 5% and decrease of LV diameters.

## Discussion

For the first time electrical delays within the CS branch system during SR and RV apical pacing are compared with aid of 3D mapping systems. The main findings of this study are that electrical delays within the CS during SR in patients with or without LBBB are highly variable and unpredictable. Of note the electrical activation in the posterior wall does not start from the apical segment, as shown also with 3D mapping [5], [6]. Secondly, RV pacing homogenizes electrical delays into a predictable parabolic distribution with greatest delays observed at 162° on the mitral ring reaching around 75% of QRS duration near the base of the heart. The considerable differences between SR and RV apical pacing with respect to regions of latest activation and electrical separation of RV and LV electrodes may have an influence on the choice for LV lead implantation. Individual mapping of CS activation in each patient is time consuming. Therefore, implanting the lead at the predicted site of latest activation during RV pacing, at 162 degrees on the mitral ring, has the greatest chance to achieve the maximal electrical separation of RV and LV lead.

Previous studies have described a heterogeneous activation of the LV in patients with LBBB [10]. Different patterns of septal activation, i.e. low vs. high septal breakthrough, have been shown to determine activation of the LV [11], [12]. Although only endocardial mapping has been performed to date, these findings match with our results of heterogeneous epicardial LV activation with interindividual differences depending possibly on septal breakthrough site, local scar and conduction system disease. In addition various activation patterns of the LV in patients with heart failure and LBBB have been demonstrated [5], [6], [13]. Thus the LBBB on the surface ECG is an incomplete characterization of intraventricular activation pattern. As a result LV activation during SR cannot be predicted and might not be useful in guiding the positioning of lead implantation. Since simultaneous RV pacing is used in most CRT patients, electrical separation to the RV lead seems preferable. When the RV lead, which is most of the time an ICD lead in patients with heart failure, is positioned near the septal RV apex, LV activation can be predicted and electrical RV-LV lead separation can be maximized based on our data.

### Rationale for a coordinate system for CS lead positions

The great variation of anatomic CS branches has led to a segmental description of CS lead positions [7]. However, anatomic nomenclature for cardiac segments varies between subspecialties: for instance the lateral segment in echocardiography [9] is posterior on fluoroscopy in the new electrophysiological nomenclature [14]. Therefore, a coordinate system is a reasonable alternative using the three standard segments in the vertical direction (apical, middle and basal segment) and a gradation of the mitral ring starting from 0° at the CS ostium in the counterclockwise direction. The turnaround point at 180° from posterior to anterior can be seen in virtually any fluoroscopic angulation, because it marks the maximal lateral position from which the electrode moves towards the RV. By visually subdividing the half circle into quarters or more, the grades on the mitral ring can be reliably estimated, as confirmed by nonfluoroscopic imaging in this study. Nonfluoroscopic imaging proved especially useful to estimate the degree of apical direction in posterior locations and is a useful tool for localising any implant electrodes, but is not mandatory for routine implantations. Systematic definitions of CS lead implant positions in further studies are preferable for statistical analysis and prospective comparisons between subjects and institutions.

### Limitations

Mechanical dyssynchrony and hemodynamic responses were not subject of the study, and therefore, were not assessed during the implant procedure. Further studies are required to correlate electrical delays within the CS to these factors. The number of electrode positions varied between patients. Therefore, we performed a weighted analysis when assessing the relationship between the electrical delay and grades on the CS ring. Nonlinear models are more difficult to fit than linear models and the results must be interpreted with caution.

### Conclusion

Electrical delays within the CS vary during SR, but prolong and become predictable during RV pacing. A coordinate system helps predicting the local delays and facilitates interindividual comparison of lead positions irrespective of CS branch anatomy and may be helpful in guiding CS lead positioning during CRT. Further studies are needed to evaluate the prognostic value of maximizing electrical separation during CRT implantation.
